# Gait-related beta-gamma phase amplitude coupling in the subthalamic nucleus of parkinsonian patients

**DOI:** 10.1038/s41598-024-57252-2

**Published:** 2024-03-20

**Authors:** AmirAli Farokhniaee, Chiara Palmisano, Jasmin Del Vecchio Del Vecchio, Gianni Pezzoli, Jens Volkmann, Ioannis U. Isaias

**Affiliations:** 1 Fondazione Grigioni Per Il Morbo Di Parkinson, Via Gianfranco Zuretti 35, 20125 Milano, Italy; 2 Parkinson Institute Milan, ASST G. Pini CTO, Via Bignami 1, 20126 Milano, Italy; 3 Department of Neurology, University Hospital of Würzburg, and Julius Maximilian University of Würzburg, Josef-Schneider-Straße 11, 97080 Würzburg, Germany

**Keywords:** Deep brain stimulation, Gait, Local field potentials, Parkinson’s disease, Phase-amplitude coupling, Subthalamic nucleus, Basal ganglia, Parkinson's disease, Data processing

## Abstract

Analysis of coupling between the phases and amplitudes of neural oscillations has gained increasing attention as an important mechanism for large-scale brain network dynamics. In Parkinson’s disease (PD), preliminary evidence indicates abnormal beta-phase coupling to gamma-amplitude in different brain areas, including the subthalamic nucleus (STN). We analyzed bilateral STN local field potentials (LFPs) in eight subjects with PD chronically implanted with deep brain stimulation electrodes during upright quiet standing and unperturbed walking. Phase-amplitude coupling (PAC) was computed using the Kullback-Liebler method, based on the modulation index. Neurophysiological recordings were correlated with clinical and kinematic measurements and individual molecular brain imaging studies ([^123^I]FP-CIT and single-photon emission computed tomography). We showed a dopamine-related increase in subthalamic beta-gamma PAC from standing to walking. Patients with poor PAC modulation and low PAC during walking spent significantly more time in the stance and double support phase of the gait cycle. Our results provide new insights into the subthalamic contribution to human gait and suggest cross-frequency coupling as a gateway mechanism to convey patient-specific information of motor control for human locomotion.

## Introduction

The supraspinal locomotor network is distributed over multiple brain areas^1–3^ and specifically wired neuronal circuits^4,5^, making its understanding a challenging task. Recent technological advances, particularly new brain stimulators capable of recording neural activity in chronically-implanted patients (e.g., AlphaDBS [Newronika], Activa PC + S^6,7^ and Percept PC [Medtronic PLC]^8^) have strongly influenced research on the control of movement and enabled the recording of neural activity during complex motor tasks, including walking^9^. Much attention has been given to patients with Parkinson’s disease (PD), due to the multiple gait problems in these patients^10^, their highly disabling impact and reduced quality of life and life expectancy^11,12^, and the lack of specific therapies. Indeed, although dopaminergic drugs^10^ and deep brain stimulation (DBS) provide sustained improvement to PD motor symptoms, their effect on gait impairment remains largely unsatisfactory^13,14^ and over 40% of PD patients with DBS of the subthalamic nucleus (STN) report subjective worsening of gait performance after surgery^15^.

The power of the beta band (~ 13–30 Hz) observed in local field potential (LFP) recordings of the STN in PD was shown to correlate with the severity of low-dopamine symptoms, i.e., bradykinesia and rigidity^16^. More recent data, however, suggest that striatal dopamine levels couple with STN oscillatory frequency^17^, rather than power modulations, as an intrinsic property of the basal ganglia circuits for proper motor performance^18,19^. This may especially apply to motor acts that involve large-scale brain networks, such as human gait^19^. Indeed, although beta power changes during walking have been reported^20–24^, this finding has not been fully confirmed^25,26^. More recently, the observation by Thenaisie et al.^27^ that activation of lower leg muscles while seated, passive leg movements, and walking along a horizontal ladder result in similar beta power modulation suggests that this may be a common aspect of motor control and not a distinctive feature of human walking.

We still know very little about the role of the STN in bipedal gait—one of the most unique and fundamental motor acts in humans^28^—but this remains crucial in order to make the best use of new implantable pulse generators^29^. To exploit the full potential of these devices, it is necessary not only to monitor symptom-specific neural signals, but also their fluctuations during activities of daily living, such as walking^26^, grasping^30^, or speech^31^, etc.

In this study we analyzed previously-acquired recordings with the Activa PC + S device (Medtronic, PLC) and computed phase-amplitude coupling (PAC) during upright standing and unperturbed walking. We then correlated PAC measures with kinematic parameters of gait and striatal dopaminergic innervation. Both are two very accurate assessments that allow more precise correlations than routinely used clinical scales.

PAC likely reflects local activity subserving network communication through cross-frequency interactions^32,33^ and is instructive in describing the correlations between the different band rhythms or frequency components present in the LFP signal. The present report complements recent studies of subthalamic activity during human gait in this^19,26,34,35^ and other^8^ cohorts.

To the best of our knowledge, no previous work has studied changes in beta-gamma PAC in patients with PD during actual walking. Only one paper, by Jin et al.^36^ described the effect of timing (with a metronome) the stepping movement while sitting in a chair, however, this paper lacks a comparison with resting state data and patients were studied 3–7 days after the surgery without withdrawal of dopaminergic medication. In any case, the authors demonstrated greater beta-gamma PAC during non-timed stepping, possibly supporting a greater involvement of the basal ganglia circuitry during internally triggered movements. Of note, in this work the authors did not describe a significant difference of beta-gamma PAC for contralateral or ipsilateral movements suggesting the need for a more refined approach involving a distinction based on laterality and severity of symptoms or, when possible, neuroimaging studies describing striatal dopaminergic denervation.

## Materials and methods

### Subjects, surgery, clinical and molecular imaging evaluation

We studied eight subjects with idiopathic PD established according to the UK Parkinson Disease Brain Bank criteria. The dataset (with different conducted analyses) has already been published in a previous article^26^. Demographic and clinical data are reported in the supplementary materials (Table S1). No patient suffered from cognitive or mood problems, as evaluated using standardized rating scales (i.e., Parkinson neuropsychometric dementia assessment, Mattis dementia rating scale, Hamilton depression rating scale, and the non-motor symptoms scale) and all had a stable and positive response to levodopa and STN-DBS (> 30% change in the Unified Parkinson’s Disease Rating Scale (UPDRS) part III score) (see^26^).

All patients were implanted with the Activa PC + S system and quadripolar macroelectrodes (model 3389, Medtronic, PLC). The precise localization of the active and recording contacts in the STN was confirmed by image fusion of the preoperative stereotactic MRI and postoperative CT scans (SureTune™, Medtronic, PLC). The surgical procedure has been previously described^37^.

Striatal dopamine reuptake transporters (DAT) density was assessed at rest using single-photon computed tomography (SPECT) and [^123^I]N-ω-fluoropropyl-2β-carbomethoxy-3β-(4-iodophenyl)nortropane ([^123^I]FP-CIT). SPECT data acquisition and analysis have been described previously^26,38^. Based on the non-displaceable binding potential (BP_ND_) of striatal DAT, we identified the STN of the brain hemisphere with less (−) or more ( +) dopamine.

The local Institutional Review Board of the University Hospital of Würzburg approved the study and all patients gave written informed consent according to the Declaration of Helsinki.

### Protocol and biomechanical assessment

Gait recordings were performed in the morning with more than 12 h withdrawal of all dopaminergic medications and off stimulation condition from at least 2 h. Subjects walked barefoot at their preferred speed over a 10 m walkway (see supplementary material, Fig. S1). At the beginning of each walking trial, we recorded about 30 s of quiet upright standing (S). Patients performed at least three walking (W) trials (range three to six) according to their clinical condition. The gait cycle was assessed with an optoelectronic system (SMART-DX, BTS) and 29 spherical retro-reflective markers^39,40^ and three Inertial Measurements Units (IMU, Opal, APDM) placed on the sternum and outer anklebones^35,41^. Gait trials were video recorded with two cameras (VIXTA, BTS). We also recorded surface muscle activity (FREEEMG, BTS) of the tibialis anterior, soleus, gastrocnemius, biceps femoris, and vastus lateralis, as well as the high-density, 64-channel electroencephalogram (EEG) (MOVE, Brain Products or Sessantaquattro, OTBioelettronica). All devices were synchronized as previously described^34,35^. Electromyography (EMG) and EEG signals were used in this study only for cleaning the LFP recordings from artefacts (see below). Gait cycles were defined by means of feet marker tracks with Matlab-based custom scripts (Matlab 2019, The MathWorks, Inc), as previously described^26^. For each trial, we computed the stride duration, length, and velocity (normalized to subject’s height), as well as the stance and double-support duration (time-normalized as a percentage of the stride duration) for all strides at steady state velocity^42^. For each subject and condition, all variables were averaged over the trials. For each subject, gait cycle variables were averaged across all available strides^43,44^. For this study, we evaluated only gait trials unaffected by gait freezing or other gait disturbances (e.g., shuffling gait or festination).

### Cleaning pipeline and artifact removals using independent component analysis

We recorded subthalamic LFP with a bipolar derivation for each STN crossing the clinically most effective and chronically active contact amplified by 2000 and sampled at 422 Hz. The LFP recordings may be contaminated by different types of artifacts^8,34^. Here, we utilized independent component analysis (ICA) with fixed-point algorithm^45^ on our whole set of data, i.e., a simultaneous recording of left and right STN LFPs along with EEG and surface bipolar EMGs, to identify the most contaminating sources of noise. Prior to running ICA, noisy channels, and epochs of data with abnormal amplitude and high amount of noise in EEG and EMG recordings were deleted by visual inspection of time series and spectrogram (see supplementary material, Fig. S2). The whole recordings were bandpass filtered from 0.5 to 100 Hz using a 5th order Butterworth bandpass filter (stopband1 = 0.5 Hz, stopband2 = 100 Hz; passband1 = 1 Hz, passband2 = 95 Hz). Then we identified independent components (ICs) using fixed-point algorithm and manually removed the ICs known to be related to cardiac rhythm and movement by looking at ICs’ time series and power spectra (see supplementary material).

### Power spectral densities and spectral power

Walking trials were defined from the first to the last identified gait cycle event in continuous periods of walking. For each patient and state (S and W), all available trials were concatenated. Power spectral density (PSD) was computed using Welch’s method, with 1 s windows and 50% overlap and averaged across 100 permutations with an initial random point.

### Phase-amplitude coupling analysis

Of the several methods to compute PAC^46–50^, we applied the Kullback-Liebler method based on the modulation index (MI), which is a measure of the entropy of phase-amplitude distribution. This method has demonstrated reliable results in several studies^17,51–53^ and, with regards to our data sample, has the advantage of providing robust solutions over multiple computations. The shortest gait duration was ~ 13 s for patient wue07. Hence, PAC was calculated over 12.5 s for all patients, in either the S or W state. The clean LFPs were bandpass filtered, once in the beta band (10–30 Hz, 2 Hz steps, bandwidth 4 Hz) and once in the gamma band (40–100 Hz, 4 Hz steps, bandwidth 10 Hz). By applying Hilbert transform, the instantaneous phase and amplitude of the beta and gamma bands were extracted, and the correlation between the beta phase and gamma amplitude was computed. In more detail, for every 20 degrees interval of the instantaneous phase distribution, the entropy of the instantaneous amplitude envelope distribution was computed. The normalization of the entropy to the maximum value provided us with the MI factor. The bigger the MI, the more coupling that exists between the phase and amplitude of the two rhythms.

### Statistical analysis

The significance of the observed PAC diagrams was computed on surrogate data, independent of the calculation method^54^. There are several ways to make the surrogate data for this purpose^48^, such as temporal shift in phase or amplitude signals obtained from the raw data. For each patient and state, we divided the amplitude vector into two halves and switched the first part with the second part and recalculated the PAC, as done in Bahramisharif and coll.^55^. We computed the surrogate PAC 200 times, cutting the raw data from random points in 12.5-s epochs and averaged the results. This formed the surrogate PAC and its subtraction from the original PAC (uncorrected) provided us with the corrected PAC values^48^. This methodology removed the spurious peaks in the uncorrected PAC diagram and ensured elimination of the randomness and noise in the raw data that might be misleading in PAC computation. Finally, the individual PAC diagrams were normalized with respect to the maximum PAC value observed in one arbitrarily-selected patient (i.e., wue02). The uncorrected PAC values were compared with the surrogate PAC values in a t-test and only significant values (*p* < 0.01) were shown in the PAC diagrams. The choice of 0.01 as the significant value is to reduce the false-positive results and is a level commonly used in previous studies^56^.

To statistically quantify the PAC difference between S and W states (PAC_S_ and PAC_W_), we summed all the MIs (total corrected PAC value) and assigned that number to each patient and corresponding state (S or W), as per Eq. 1.

1 $${{\text{PAC}}}_{state}={\sum }_{k=1}^{n}{MI}_{k}$$

where *state* indicates S or W and *n* is the total number of elements present in the PAC diagram. We also evaluated the change of PAC between S and W (PAC△_SW_) and the total PAC value for the low beta band (13–20 Hz, L-PAC) and high beta band (20–30 Hz, H-PAC).

We compared the PAC_W_, PAC_S_, the L-PAC, and the H-PAC between states using paired t-test. To estimate linear correlation between kinematic or clinical data with PAC values, we utilized Pearson’s and Spearman’s correlations considering the correction for multiple comparisons.

### Ethical approval

University of Würzburg n. 103/20, n. 36/17, and n. 228/13.

### Consent to participate and publication

All authors consent to participate in this research and the publication of the data and results.

## Results

### Demographic, clinical, biomechanics and molecular imaging data

A detailed description of the demographic and clinical data, imaging, and biomechanics for each of our patients has previously been reported^19,26,35^. In summary, at the time of the study the mean age of the patients was 57 years (± 4.8 SD) and the mean disease duration was 12 years (± 4.2 SD). All patients improved after STN-DBS by more than 60% at the UPDRS-III score. The mean percentage reduction of levodopa equivalent daily dose after STN-DBS was 43.4%. In the more dopamine-depleted brain hemisphere (STN-), the reduction of DAT averaged 70.5% (± 10.1 SD), while in the other hemisphere (STN +) it was 60.2% (± 12.0 SD). The kinematic data and correlations are shown in Fig. 3.

### Power spectral densities and spectral power

At a group level, we did not find any significant power modulation between standing and walking^26,35^. However, we have noticed great variability among patients, and we consider it important to present in the supplementary material the individual patient assessment of the PSDs (Figures S3, S4) and band power distributions (Figures S5, S6).

### PAC diagrams

**Figure 1:**
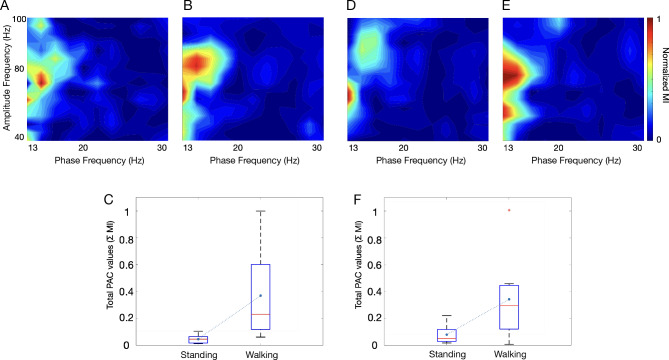
Phase-amplitude coupling (PAC) diagrams. Mean PAC values with STN- or STN + in standing (**A** and **D**, respectively) and walking (**B** and **E**) and boxplots (**C** and **F**) for total PAC during the two conditions (*p* = 0.002 and *p* = 0.003, respectively, Kruskal–Wallis test). The boundaries of the box plots indicate the 25 and 75 percentiles of the distributions, and the red lines show their median. The means of the distributions were added as blue-filled circles. The red plus sign indicates the outlier of the distribution. STN, subthalamic nucleus (+ / − , more or less dopaminergic innervation).

**Figure 2:**
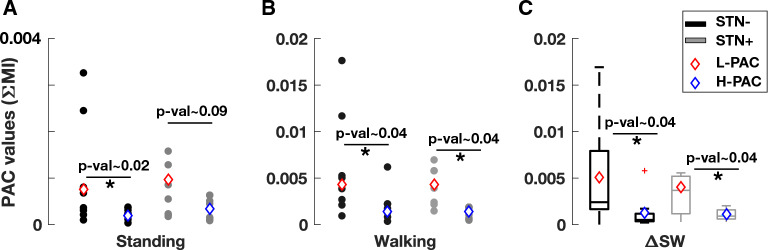
Comparison between H-PAC and L-PAC. Distributions of the L-PAC and H-PAC values during standing, walking, and their difference for both STN- and STN + groups, shown by boxplots. The boundaries of the box plots indicate the 25 and 75 percentiles of the distributions, and the black/gray lines show their median. The means of the distributions were added as red/blue diamonds. The red plus signs indicate the outliers of the distributions. Significant *p*-values (using paired t-test) are marked by an asterisk. H- or L-PAC, total phase-amplitude coupling value for the high or low beta band; STN, subthalamic nucleus (+ / − , more or less dopaminergic innervation).

We evaluated the mean behavior of PAC changes for each patient by taking the average of all PAC diagrams for S and W states for both STN- and STN + (see Fig. S7 for individual PAC diagrams in S and W) and found a significant increase in PAC between the two states (Fig. 1) in both STN- (Fig. 1A–C) and STN + (Fig. 1D–F). Statistical significance (utilizing paired t-test) was achieved for H-PAC and L-PAC, but lower frequencies showed more predominant PAC modulation (Fig. 2).

### Correlation between PAC measurements and clinical and biomechanical data

Clinical severity measured with UPDRS-III scores did not correlate with PAC_S_ (STN- r = −0.32, *p* = 0.22; STN + r = 0.51, *p* = 0.1), PAC_W_ (STN- r = −0.27, *p* = 0.26; STN + r = 0.23, *p* = 0.29) and with PAC△_SW_ (STN- r = −0.24, *p* = 0.28; STN + r = 0.10, *p* = 0.59).

**Figure 3:**
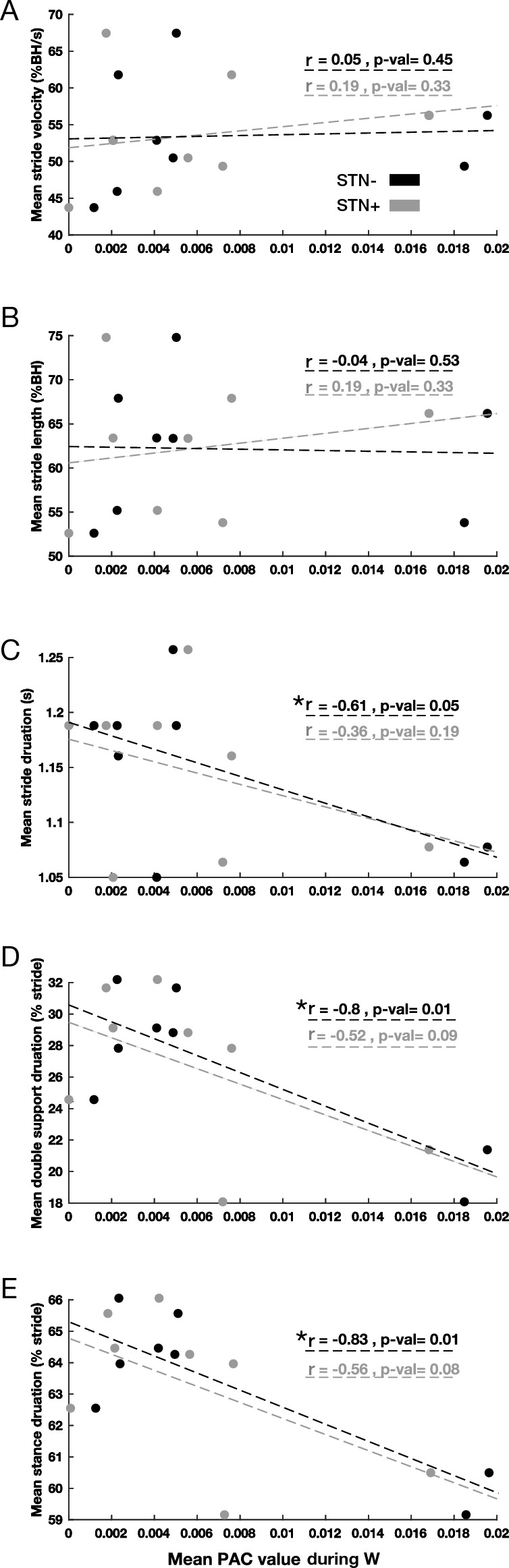
Kinematics and PAC_W_ correlations. Correlation diagrams between PAC values of STN + and STN- in patients during the walking state and (**A**) mean stride velocity, (**B**) mean stride length, (**C**) mean stride duration, (**D**) mean double support duration, and (**E**) mean stance duration. Significant correlations are marked by an asterisk for *p* < 0.05 and two asterisks for *p* < 0.01. PAC, phase-amplitude coupling; S, standing; W, walking; STN, subthalamic nucleus (+ / − , more or less dopaminergic innervation).

**Figure 4:**
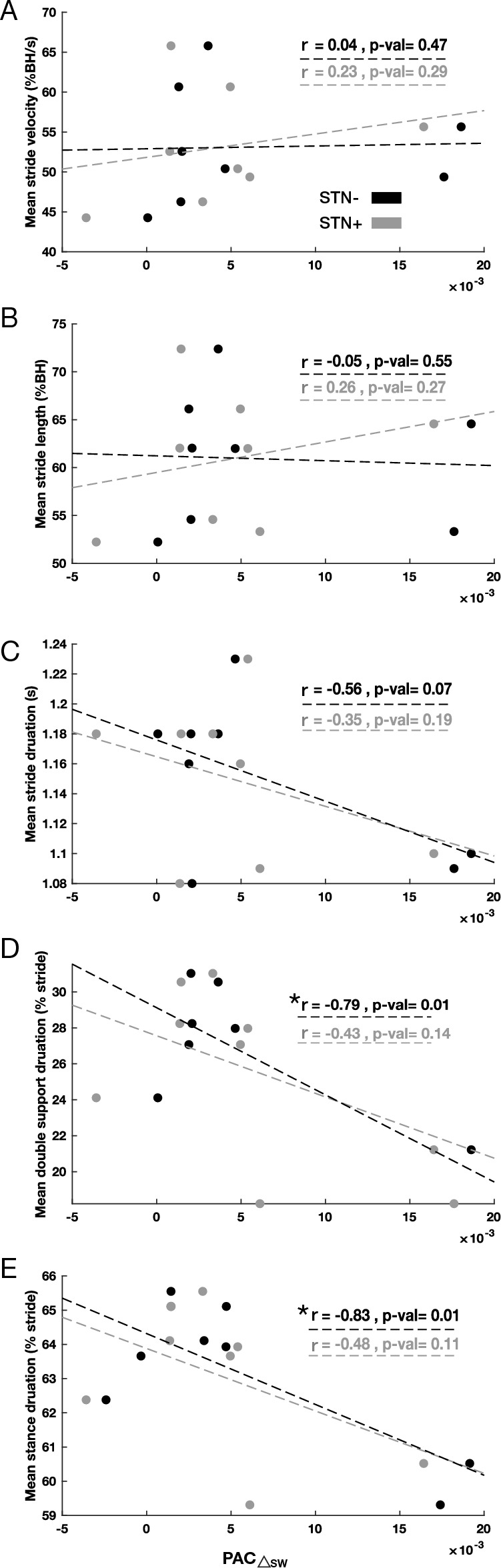
Kinematics and PAC△_SW_ correlations. Correlation diagrams between change in PAC values of STN + and STN- in patients and (**A**) mean stride velocity, (**B**) mean stride length, (**C**) mean stride duration, (**D**) mean double support duration, and (**E**) mean stance duration. Significant correlations are marked by an asterisk for *p* < 0.05 and two asterisks for *p* < 0.01. PAC, phase-amplitude coupling; S, standing; W, walking; STN, subthalamic nucleus (+ / − , more or less dopaminergic innervation).

In the more dopamine-depleted brain hemisphere (STN-), low PAC values in walking and low PAC changes between conditions (PAC△_SW_) resulted in more time spent in the weight support phase (stance or double support) of the gait cycle (Figs. 3 and 4). Stance duration and double support duration correlated selectively with PAC measurements of the STN- (Figs. 3 and 4). Nevertheless, these results were carried out by estimating the Pearson correlation. In addition, we conducted the Spearman correlation to check the non-linear correlation of the same values and we did not find any significant correlation.

### Correlation between PAC measurements and BP_ND_

**Figure 5:**
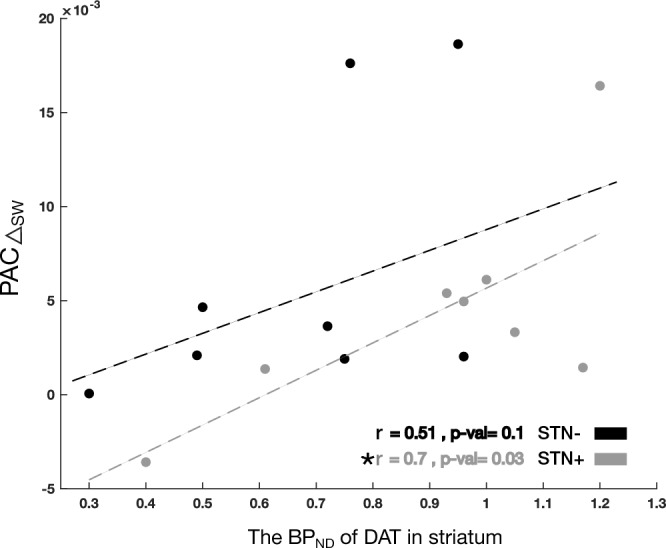
Correlation between changes in phase-amplitude coupling (PAC) values and the corresponding non-displaceable binding potential (BP_ND_) of dopamine reuptake transporters (DAT) of patients. The STN + group (i.e., more dopaminergic innervation in the subthalamic nucleus) showed a significant correlation, marked by an asterisk, *p* < 0.05.

In the less dopamine-depleted brain hemisphere (STN +), the more striatal dopamine, the more the PAC increased during walking (Fig. 5). This result was confirmed by a separate evaluation of BP_ND_ of the caudate nucleus and the putamen (Table 1). For STN- we did not find any correlation between PAC measurements and BP_ND_ (Table 1). These results were obtained by estimating Pearson correlation, however, for the case of Fig. 5, we also estimated the Spearman correlation on the same data and found that there is a significant correlation (r = 0.6, *p* = 0.05) in STN + .

**Table 1:** Correlations between PAC values of the STN + and STN- and the BP_ND_ of DAT given as Pearson’s correlation r and the corresponding *p*-values.

STN +	Striatum BP_ND_	Putamen BP_ND_	Caudate nucleus BP_ND_
PAC_W_	0.61, *p* = 0.05*	0.61, *p* = 0.05*	0.63, *p* = 0.04*
PAC_S_	−0.65, *p* = 0.04*	−0.64, *p* = 0.04*	−0.62, *p* = 0.05*
PAC△_SW_	0.7, *p* = 0.03*	0.7, *p* = 0.03*	0.71, *p* = 0.02*

STN-	Striatum BP_ND_	Putamen BP_ND_	Caudate nucleus BP_ND_
PAC_W_	0.48, *p* = 0.12	0.36, *p* = 0.19	−0.53, *p* = 0.09
PAC_S_	−0.37, *p* = 0.18	−0.45, *p* = 0.13	−0.31, *p* = 0.23
PAC△_SW_	0.51, *p* = 0.1	0.39, *p* = 0.17	0.56, *p* = 0.08

*Significant correlations. BP_ND_, non-displaceable binding potential; DAT, dopamine reuptake transporters; PAC, phase-amplitude coupling; S, standing; STN, subthalamic nucleus (+ /−, more or less dopaminergic innervation); W, walking.

## Discussion

We have shown an increase in subthalamic beta-gamma PAC between standing and walking in PD. Patients with poor PAC modulation during walking spent more time in the weight-bearing (stability) phase of the gait cycle. Striatal dopamine promotes gait-related cross-frequency coupling in the STN of parkinsonian patients.

Human walking is a complex motor behavior that requires timed coordination of several cortical and subcortical brain areas^1–3^. The STN is a key node of the supraspinal locomotor network that is directly connected to the supplementary motor and parietal areas and projects to the mesencephalic locomotor region^57,58^. In recent years, technological advances^29^ have enabled important information to be obtained about the composite subthalamic dynamics of human locomotion^9,13^. Some studies reporting basal ganglia field potential recorded from implanted DBS leads in patients with PD showed modulation of beta oscillations during stepping and actual gait^21–24^. Still, the analysis of power spectral modulation might not fully capture the complex large-scale network dynamics of gait control^25,26^, which eventually encompass multiple frequencies^19^ to timely direct the information flow across distant brain areas^32,59–62^. Indeed, there is growing evidence that frequency modulation conveys information about movement execution in a patient-specific and frequency-related manner^18,19,30,61,63^, and that dopamine deficiency results in impaired encoding of this information^18,30^.

As a special case of cross-frequency coupling, PAC is the modulation of the amplitude of high‐frequency oscillations by the phase of low-frequency ones and represents information processing and transmission across brain areas that are involved in multiple activities, such as cognition, perception, and movement^33,46,51,64,65^. The entrainment of oscillatory activity in one frequency band according to the phase of another frequency band has been proposed to be a gateway mechanism to selectively allow task-relevant inputs to be processed^59,66–71^. Low-frequency oscillations would act as a carrier^19^ that coordinates neural activity of local and remote brain region for long-range communication through frequency and amplitude modulation^30,72^. This has been shown in the hippocampus, for example, to organize the readout from long-term memory of the discrete sequence of upcoming places, as cued by current position^73^. Similarly, we envision a key role for the STN, where beta phase-coding would be a mechanism to selectively allow task-relevant inputs to be processed and waived through to basal ganglia for an action release (gamma rhythm)^74,75^. Interestingly, low beta frequencies may be involved in the keying of a task or behavior, while high beta frequencies would carry information about its execution^30^. In line, we demonstrated a higher PAC in low beta band during a task, i.e., walking, which in this study did not involve any modulation (change in its execution) being linear and unperturbed. This interesting perspective needs further specific investigation.

In PD, finely-tuned gamma oscillations (60–90 Hz) are prokinetic network phenomena^72,76^ that increase during voluntary movements^74,77^ and correlate positively with movement velocity^78^. Previous studies in PD identified excessive beta-phase coupling to broadband high-gamma amplitude in STN^83^, M1^79^ and STN phase-M1 amplitude^56,67^. This was associated with the parkinsonian motor state and was modulated by dopaminergic medication and DBS therapy^56,79,80^. As for beta power modulation, however, these results are read on the understanding of a direct link between beta oscillations and akinetic-rigid symptoms, without allowing for the physiological or compensatory contribution of these signals^30,81^. Similar reasoning can be applied to freezing of gait^82^, where it is necessary to distinguish the actual episode of gait freezing from the component of standing while freezing, and attempting to overcome the freezing episode, as well as the physiological neural activity related to gait modulation^35^. This aspect has not yet been studied in a precise and standardized method^42^.

Our data would favor the hypothesis of a physiological contribution of subthalamic beta-gamma modulation to human gait or a compensatory activity based on the residual dopaminergic availability to promote locomotion in parkinsonian patients. This partly derives from the fact that all subjects enrolled showed substantially normal and symmetric gait kinematic measures (see^26^) and from the fact that poor PAC modulation results in a longer time in the stance (weight-bearing) phase of the gait cycle, indicating more time and effort for PD patients for posture stabilization^83^. In this regard, the correlation between PAC△_SW_ and striatal DAT density selectively in the less dopamine-impaired hemisphere (Fig. 5) may suggest a threshold effect related to an imbalance of dopaminergic activity between the two hemispheres^26,84^. The impact of (patient-specific) compensatory mechanisms may account also for the great variability in results when analyzed at single patient level in this study and in previous works.^85,86^ In some cases (e.g., wue09_R) it is possible to detect subthalamic frequency shift with no change in beta power, with an increase in power (i.e., wue04_L and wue07_R) or with its reduction and the emergence of a dual peak during walking (i.e., wue11_L). Future studies on more patients should try to identify the functional correlate of this different and multifaced neural activity.

A major limitation of our study is that we have not described a causal or exclusive relationship between increased subthalamic beta-gamma PAC and gait. Another limitation of the study is the low number of patients. This effected the utilization of Pearson and Spearman measures for correlation between biomechanical data and PAC values (Figs. 3 and 4). More specifically, the low number of patients may result in non-significant Spearman (nonlinear) correlations, but also significant Pearson (linear) correlations, that will need to be investigated in future studies with a larger number of participants. We were also unable to define the origin of beta-gamma PAC in the STN of parkinsonian patients. de Hemptinne and coll. have proposed that this activity results from the organization of STN spikes into synchronized bursts, with a short interval within the bursts^56^; however, comparison of PAC and spike-phase locking values have shown no correlations^87^. It would also have been very interesting to evaluate beta-gamma PAC between the STN and cortical areas; however, in our study we aimed to explore mainly the contribution of the STN to the parkinsonian gait. Reliable subthalamic input signals (which may include PAC^88^) that code for gait are still an unmet need when using novel stimulators in adaptive mode while preventing patients from additional implants. In this study, cortical EEG were used (see Methods) to obtain trustworthy subthalamic LFPs given the possible artifact contamination^8,22,34^. Further studies will investigate the role of different cortical areas and the interesting cortico-STN interactions during human walking. The Activa PC + S device gave us the possibility of performing recordings in chronically-implanted patients, reducing the impact of the stun effect. However, this added an additional variable to consider, which was chronic treatment with DBS (in patients also stimulated for years with dopaminergic drugs) may result in long-term effects. The “exhaustion” of these effects was partly monitored in our studies by a return to symptom severity after turning the stimulator off, comparable to the pre-implantation assessment (see^26^).

Being able to understand the contribution of specific brain areas such as the STN in the context of complex motor behaviors remains one of the frontiers of neuroscience. The difficulty of coding the physiological, compensatory, and pathological aspects of human gait can be perceived from the fact that we have no effective pharmacological or neuromodulation treatment for gait disorders. We are hopeful that advances in technology will allow us to collect more and better data, even in an ecological context, to learn more about bipedal walking—a simple yet complex motor act that defines our species^28^.

### Supplementary Information

Supplementary Information.

## Data Availability

The data are available for researchers upon reasonable request to the corresponding author, farokhniaee@parkinson.it.

## Abbreviations

[^123^I]FP-CIT: [^123^I]N-ω-fluoropropyl-2β-carbomethoxy-3β-(4-iodophenyl)nortropane
BP_ND_: Non-displaceable binding potential
DAT: Dopamine reuptake transporters
DBS: Deep brain stimulation
EEG: Electroencephalogram
EMG: Electromyography
H-PAC: Total phase-amplitude coupling value for the high beta band
ICA: Independent component analysis
IC: Independent component
L-PAC: Total phase-amplitude coupling value for the low beta band
LFP: Local field potential
MI: Modulation Index
PAC: Phase-amplitude coupling
PD: Parkinson’s disease
S: Standing
PSD: Power spectral density
SPECT: Single-photon computed tomography
STN: Subthalamic nucleus (+ /-, more or less dopaminergic innervation)
UPDRS: Unified Parkinson’s disease rating scale
W: Walking

## Code availability

The codes are available for researchers upon reasonable request to the corresponding author, farokhniaee@parkinson.it.
