# Supplementary material for: Gait-related beta-gamma phase amplitude coupling in the subthalamic nucleus of parkinsonian patients
Source: Sci Rep. 2024 Mar 20;14:6674. doi: 10.1038/s41598-024-57252-2 (PMC10954750; doi:10.1038/s41598-024-57252-2)

**SUPPLEMENTARY MATERIAL**

***for***

**Gait-related beta-gamma phase amplitude coupling in the subthalamic nucleus of parkinsonian patients**

AmirAli Farokhniaee*^a,b^, Chiara Palmisano^c^, Jasmin Del Vecchio Del Vecchio^c^, Gianni Pezzoli^a,b^, Jens Volkmann^c^, and Ioannis U. Isaias^b,c^

^a^ Fondazione Grigioni per il Morbo di Parkinson, Via Gianfranco Zuretti 35, 20125 Milano, Italy

^b^ Parkinson Institute Milan, ASST G. Pini CTO, via Bignami 1, 20126 Milano, Italy

^c^ Department of Neurology, University Hospital of Würzburg, and Julius Maximilian University of Würzburg, Josef-Schneider-Straße 11, 97080 Würzburg, Germany

*** Correspondence:** farokhniaee@parkinson.it

Table S1 contains demographic and clinical data of the recruited patients. Figure S1 contains introductory diagrams to the experimental setup and analysis workflow utilized in this paper. An example of the cleaning pipeline is shown in Figure S2. The total power spectral densities (PSDs) for each patient and state were computed and presented for STN- (Figure S3) and STN+ (Figure S4) groups. We also determined the band power distributions in different rhythms for the STN- (Figure S5) and STN+ (Figure S6) groups. The individual PAC diagrams in the standing and walking states are shown in Figure S7. PAC, phase-amplitude coupling; STN, subthalamic nucleus (+/-, more or less dopaminergic innervation).

|  | | | | | **UPDRS-III**  **pre-surgery** | | **UPDRS-III**  **post-surgery** | | | |
| --- | --- | --- | --- | --- | --- | --- | --- | --- | --- | --- |
| **ID** | **sex** | **age (yrs)** | **disease duration (yrs)** | **Δ LEDD pre-post surgery (%)** | **meds-OFF** | **meds-ON** | **meds-OFF stim-OFF** | **meds-OFF stim-ON** | **meds-ON stim-OFF** | **meds-ON stim-OFF** |
| **wue02** | M | 65 | 10 | -27.27 | 40 | 23 | 39 | 19 | 17 | 16 |
| **wue03** | M | 61 | 18 | -77.98 | 40 | 9 | 45 | 17 | 23 | 14 |
| **wue04** | M | 54 | 7 | -39.21 | 26 | 8 | 27 | 5 | 9 | 8 |
| **wue06** | M | 51 | 11 | -84.11 | 46 | 11 | 48 | 12 | 11 | 6 |
| **wue07** | M | 61 | 10 | -66.15 | 43 | 24 | 29 | 15 | 8 | 9 |
| **wue09** | M | 55 | 19 | -39.17 | 50 | 11 | 33 | 16 | 8 | 11 |
| **wue10** | M | 56 | 10 | -54.17 | 69 | 14 | 65 | 25 | 20 | 5 |
| **wue11** | F | 53 | 11 | -64.62 | 55 | 4 | 51 | 9 | 13 | 14 |

**Table S1.** Demographic and clinical data of the recruited patients. Abbreviations: LEDD: Levodopa Equivalent Daily Dose; Δ LEDD: percentual variation in the LEDD after the surgery (LEDD_before the surgery_ – LEDD_after the surgery_)*100/ LEDD_before the surgery_); UPDRS-III: Unified Parkinson’s Disease Rating Scale part III.

**Figure S1.** **A)** The Experimental setup. On the left, the schematic patient is shown with high density electroencephalogram (EEG) cap and electrodes along with implantable pulse generator (IPG) for deep brain stimulation (DBS), accompanying wires and implanted electrodes in the brain to record local field potential (LFP), accompanied by electromyogram (EMG) probes utilized for monitoring cardiac activity and synchronization purposes. The middle and right images provide frontal and rear view, respectively, detailing the precise positioning of markers and EMG probes of the experimental setup. **B)** An overview of the analysis procedure. Utilizing LFP, EMG, and EEG data, Independent Component Analysis (ICA) was employed to clean LFP signals from contaminating artifacts. Walking epochs were precisely delineated from standing periods using tracked markers. Spectral and phase-amplitude coupling (PAC) analyses were performed on both states, followed by statistical analysis and correlations described in the main manuscript.

***Independent component analysis (ICA) for artifact removal***

We removed contaminating independent components (ICs) from the original recorded LFP to obtain a clean LFP by considering the whole pack of simultaneous recordings (EEG+LFP+EMG+ECG). Note that EMG and ECG signals were present only for the walking state. Figure S2 A shows the original simultaneous EEG, LFP and EMG recordings in patient wue02 during walking in a trial. Figure S2 B shows the ICs for the example shown in A. We present the selection criteria in Figure S2 C, i.e., looking at the ICs time series and spectra simultaneously to decide for the ones to be removed. Figure S2 D shows the result of the decision on the right STN LFP, by plotting the original data (black) and so-called cleaned data (red).

***Power Spectral Densities***

The total PSDs for each patient and state were computed and the results are presented in Figures S3 and S4 for each STN- and STN+ group, respectively. For each of the subplots (top and middle rows) in Figures S3 and S4, the PSD of 100 epochs for 20 seconds are computed (dashed magenta for standing and dashed cyan for walking) and the average of these 100 trials are plotted in a thick line (red for standing, blue for walking). They are normalized to the total integral of the PSD. Note that this is done on the concatenated LFPs. The subplots at the bottom, are repetitions of all the patients mean PSDs during standing and walking, red and blue (dashed), respectively and their overall mean (thick red and blue lines).

***Band power distributions in different rhythms***

We computed the alpha (5-12 Hz), beta (13-30 Hz)) and gamma (40-100 Hz) powers (normalized with respect to the total LFP power from 5 to 100 Hz) and plotted their distributions. In addition, we divided the beta band to low (13-20 Hz) and high bands (21-30 Hz) to check if there is a change in smaller ranges of beta band rhythm. In non of the cases a significant difference between the mean of the standing and walking state distributions was seen running Kruskal-Wallis test. See Figure S5 for STN- group and Figure S6 for STN+ group of the LFPs.

****Figure S2.** A) EEG, EMG and LFP data (both left and right STN nuclei) of patient wue02 during a walking trial are plotted together, the last two time-series at the bottom are LFPs. B) ICA was run on the data shown in A and the first 20 ICs are plotted. Clearly the first IC from the top is a source of contamination. C) we take a deeper look at the first 5 ICs’ time series and power spectra to identify the contaminating ICs more precisely. In this example, IC1 (the first IC) is a source of contamination, as the time series has an odd pattern and its corresponding power spectrum (the red curve) shows no dominant activity peak particularly at frequencies lower than 20 Hz and constantly increasing, which means it is not originated form a brain source. D) the original and cleaned LFP data after ICA application for the patient’s right STN LFP.


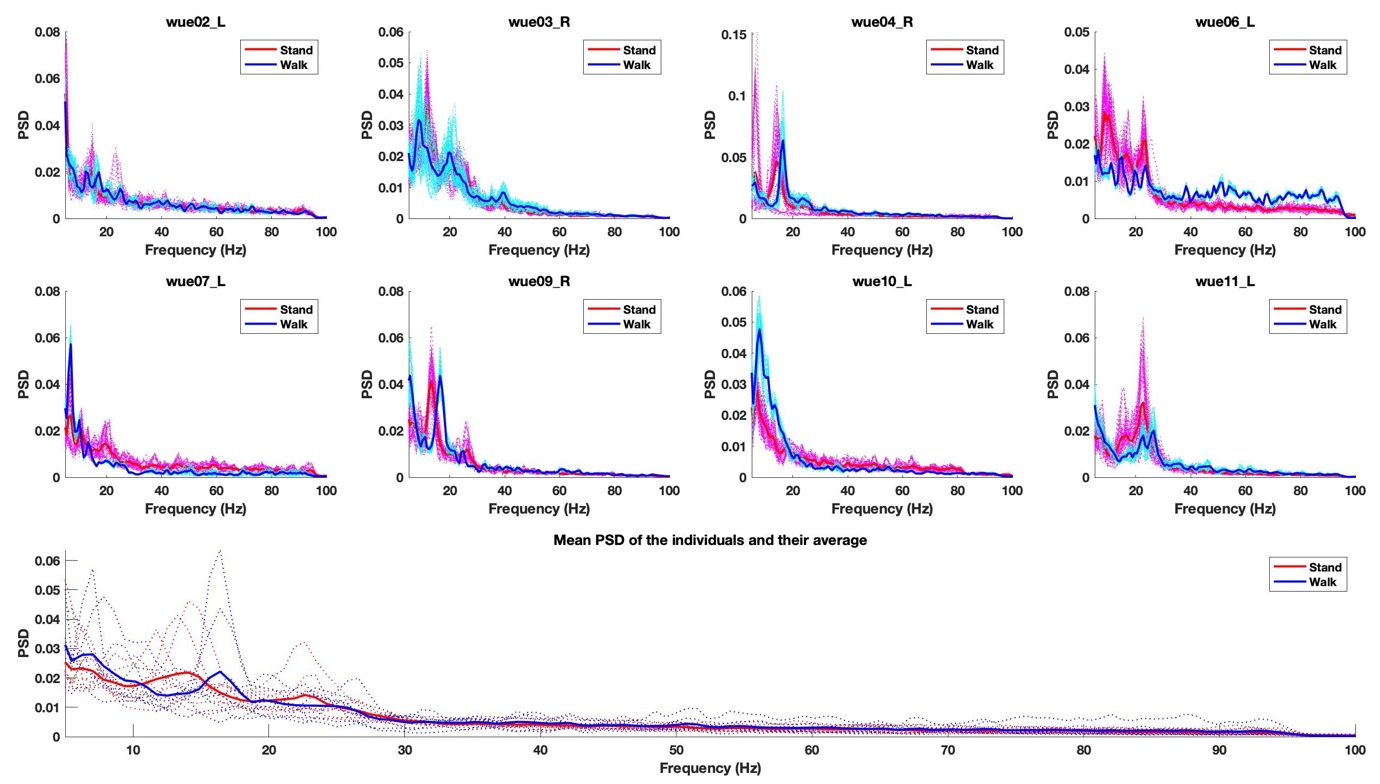


**Figure S3.** Top and middle rows: The PSDs for individual subjects in STN- group; light-dotted lines are each single walking (cyan) or standing (magenta) trial and the bold line their average (red for standing and blue for walking). Bottom row: In bold, the grand average of PSDs for STN- LFPs over all patients (red for standing and blue for walking) and light-dotted the average for each individual over its numerous trials.


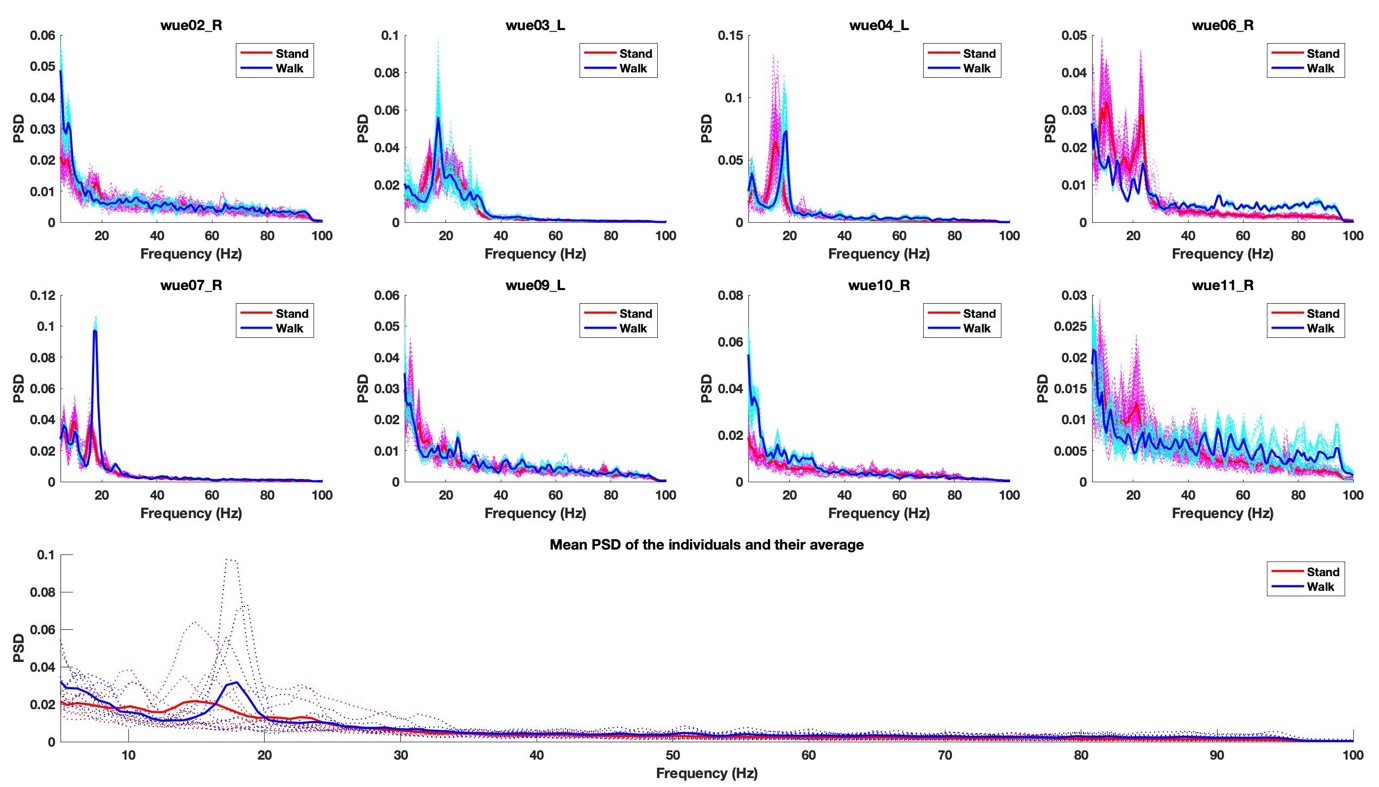


**Figure S4.** Top and middle rows: The PSDs for individual subjects in STN+ group; light-dotted lines are each single walking (cyan) or standing (magenta) trial and the bold line their average (red for standing and blue for walking). Bottom row: In bold, the grand average of PSDs for STN+ LFPs over all patients (red for standing and blue for walking) and light-dotted the average for each individual over its numerous trials.

**Figure S5.** A) alpha (p = 0.17), B) beta (p = 0.4), C) low-beta (p = 0.46), D) high-beta (p = 0.3), and E) gamma band power (p = 0.6) distributions during standing and walking states for STN- group.

******

**Figure S6.** A) alpha (p = 0.67), B) beta (p = 0.75), C) low-beta (p = 0.46), D) high-beta (p = 0.29), and E) gamma band power (p = 0.34) distributions during standing and walking states for STN+ group.

**Figure S7.** Individual PAC diagrams in standing and walking states:

***WUE02***


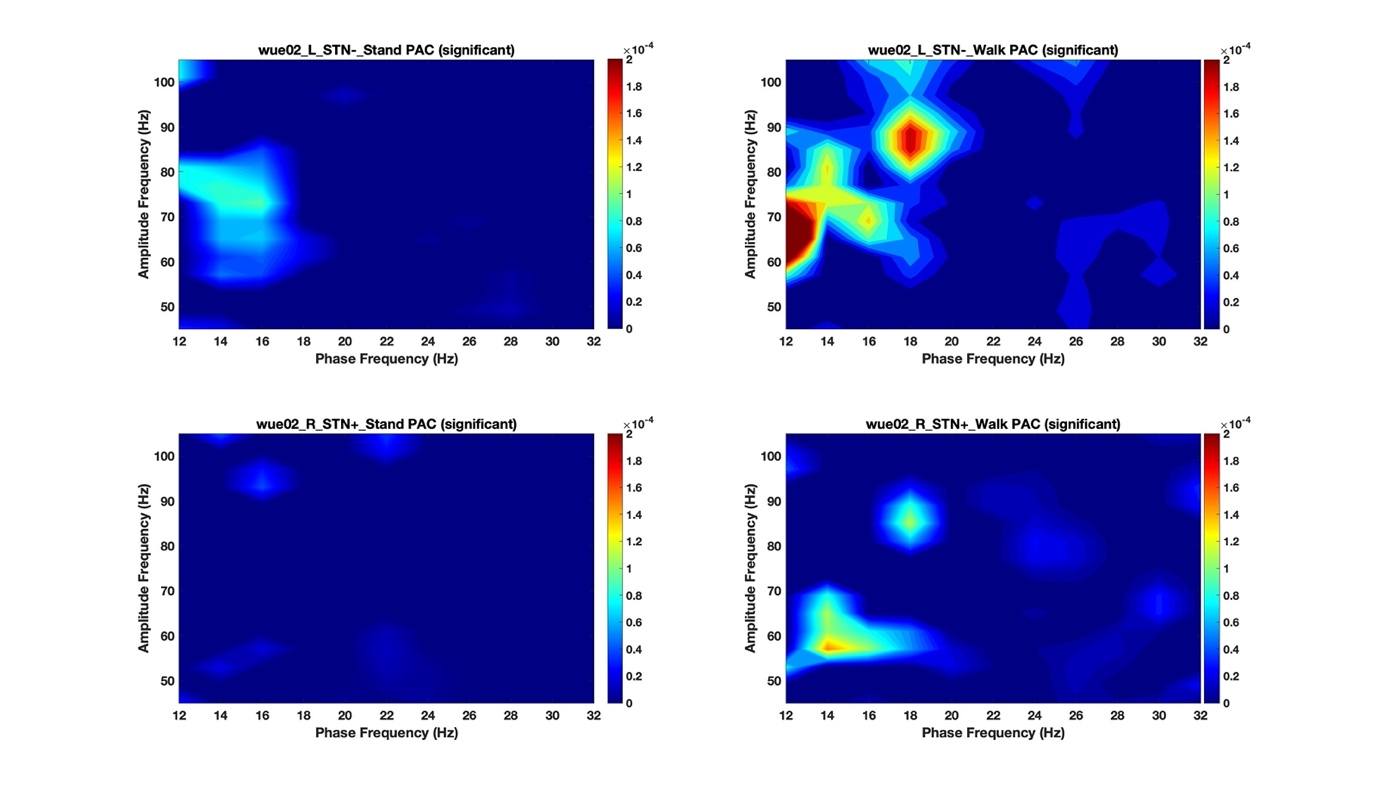


***WUE03***


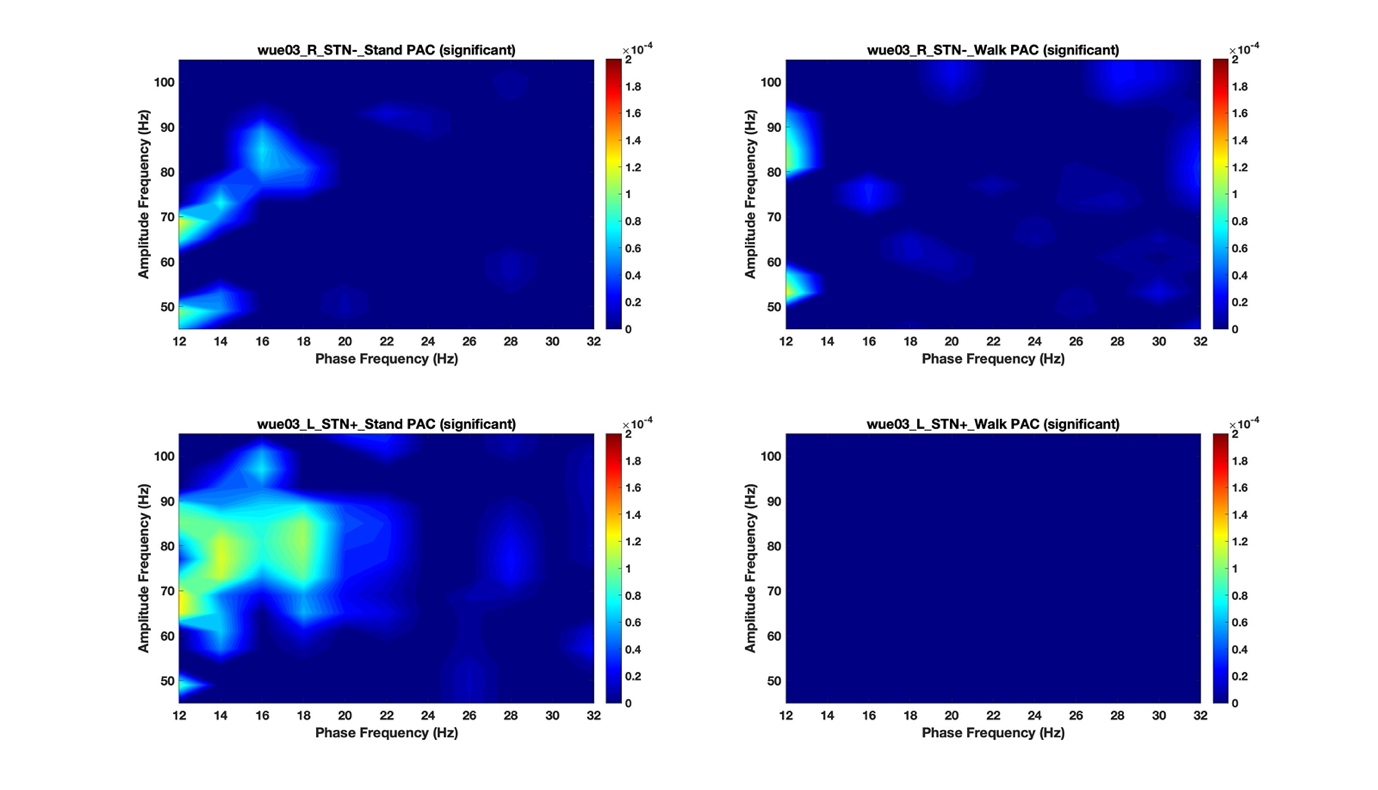


***WUE04***


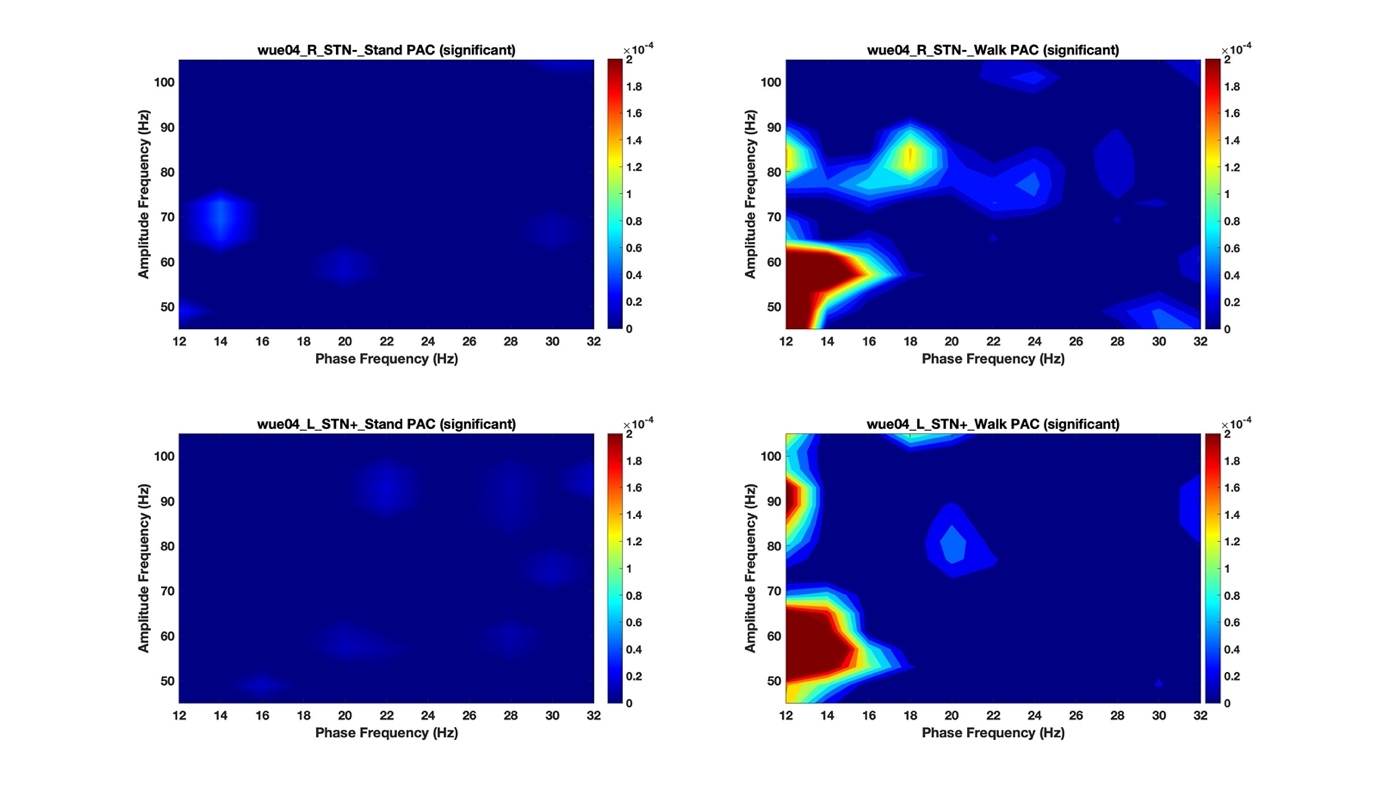


***WUE06***


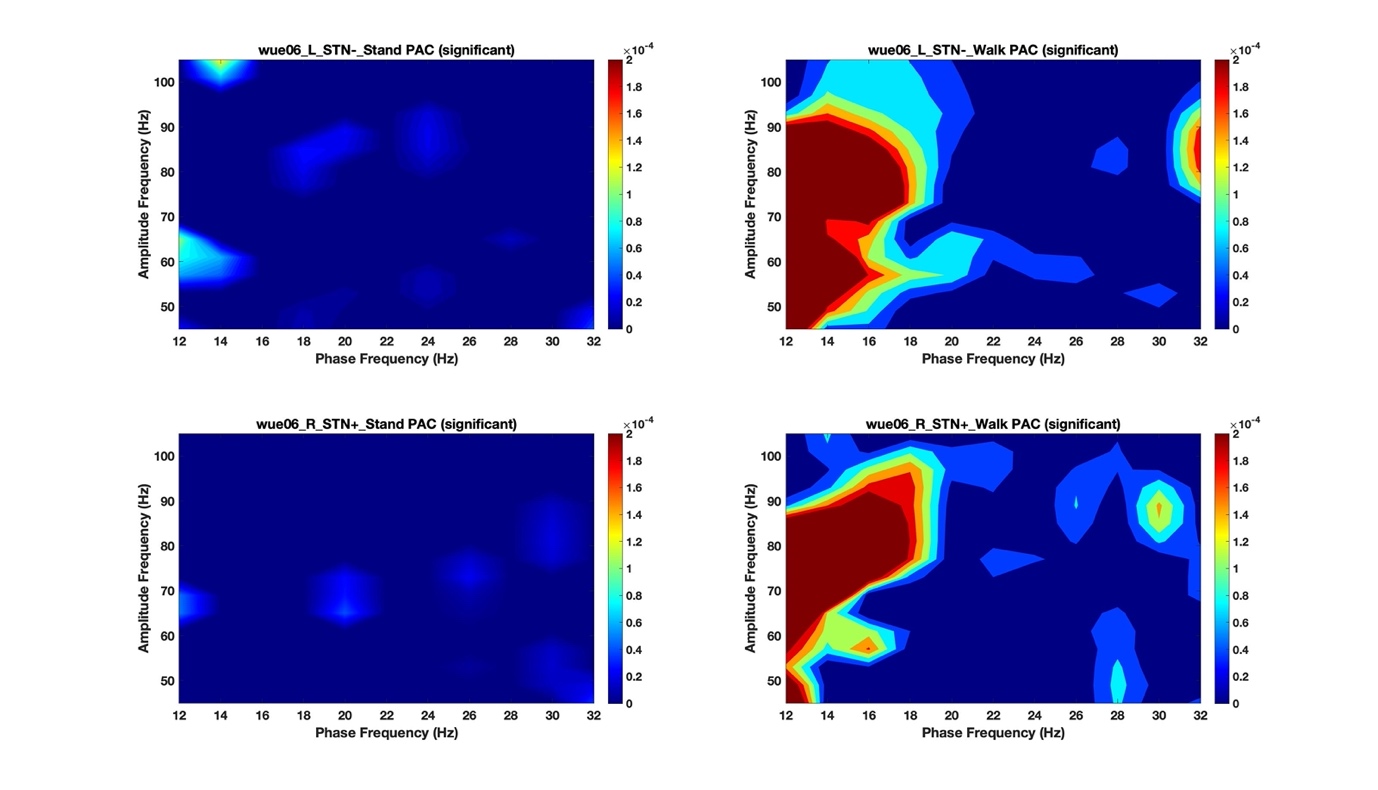


***WUE07***


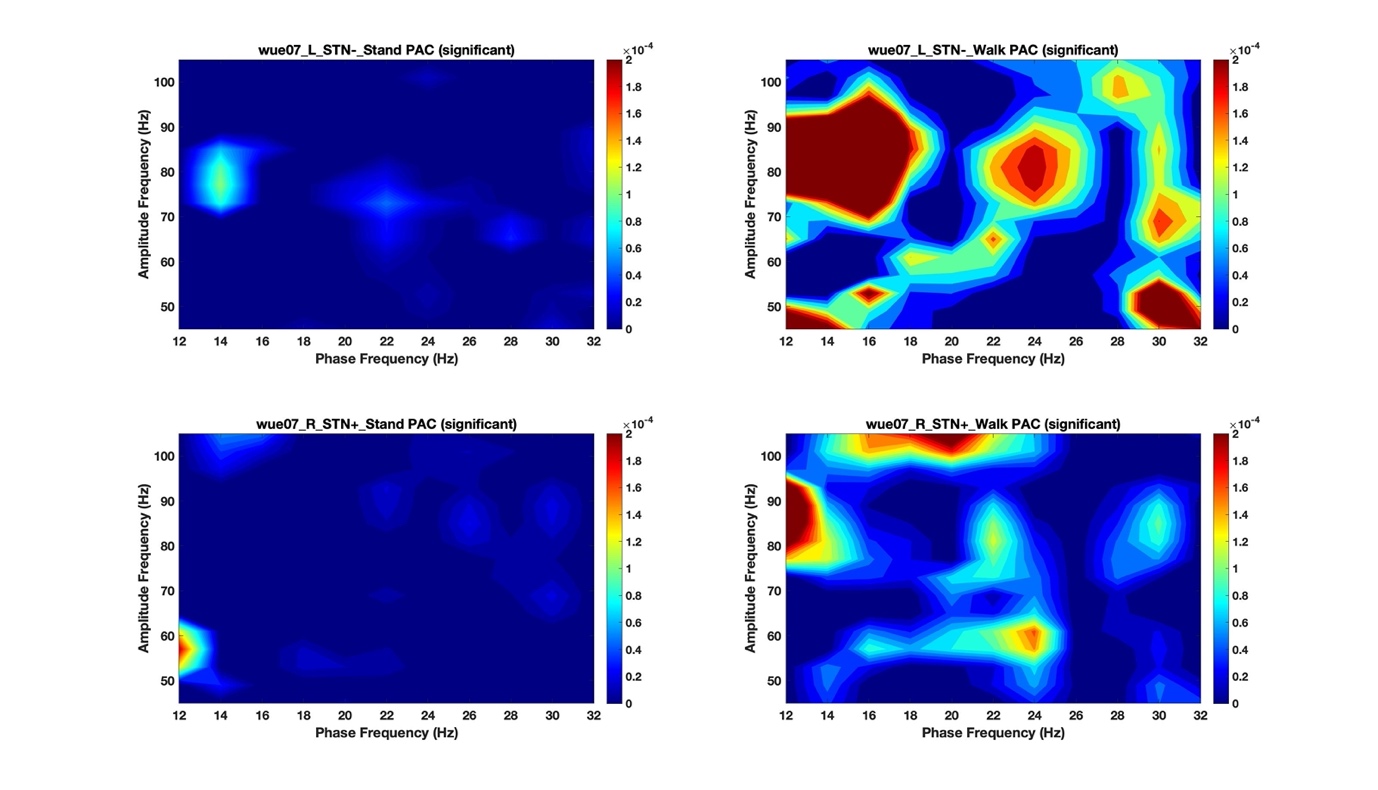


***WUE09***


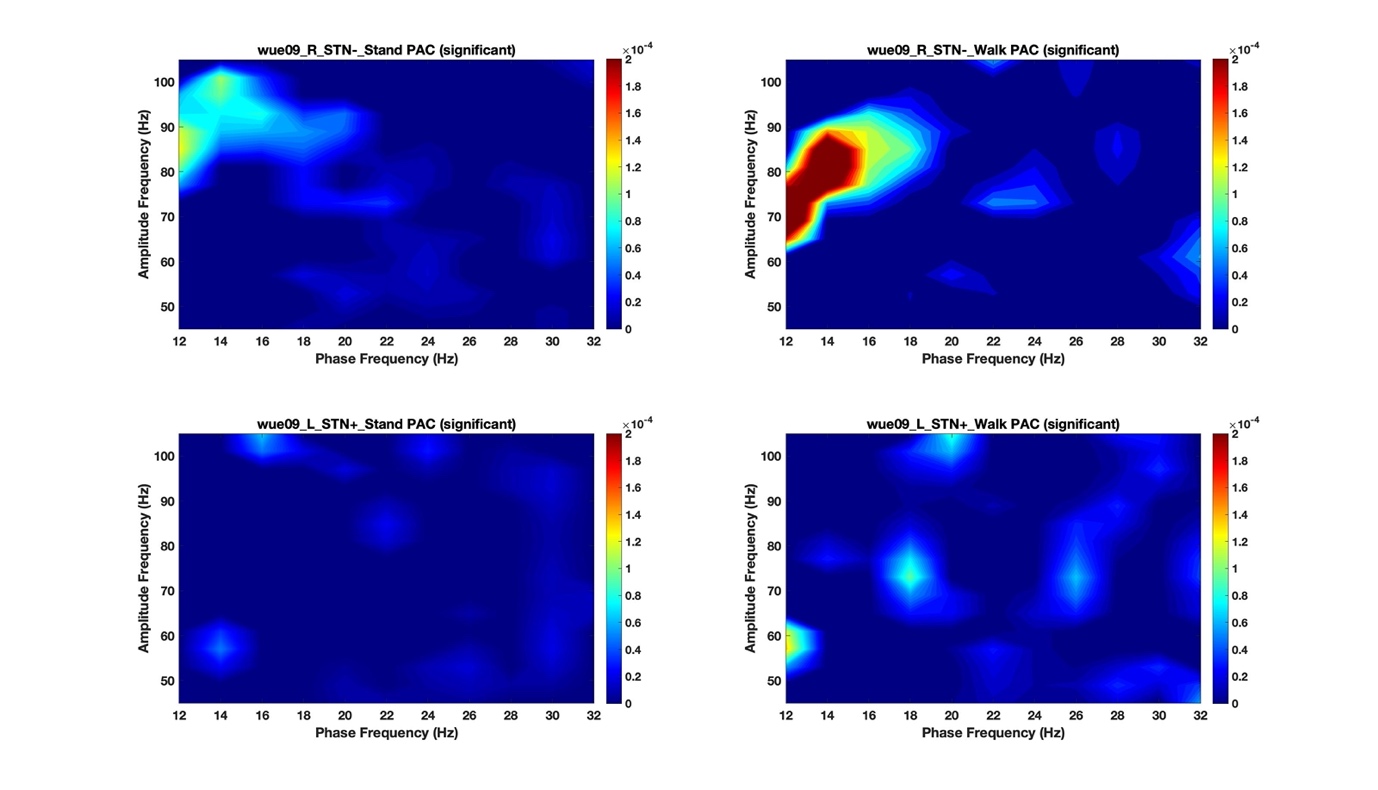


***WUE10***


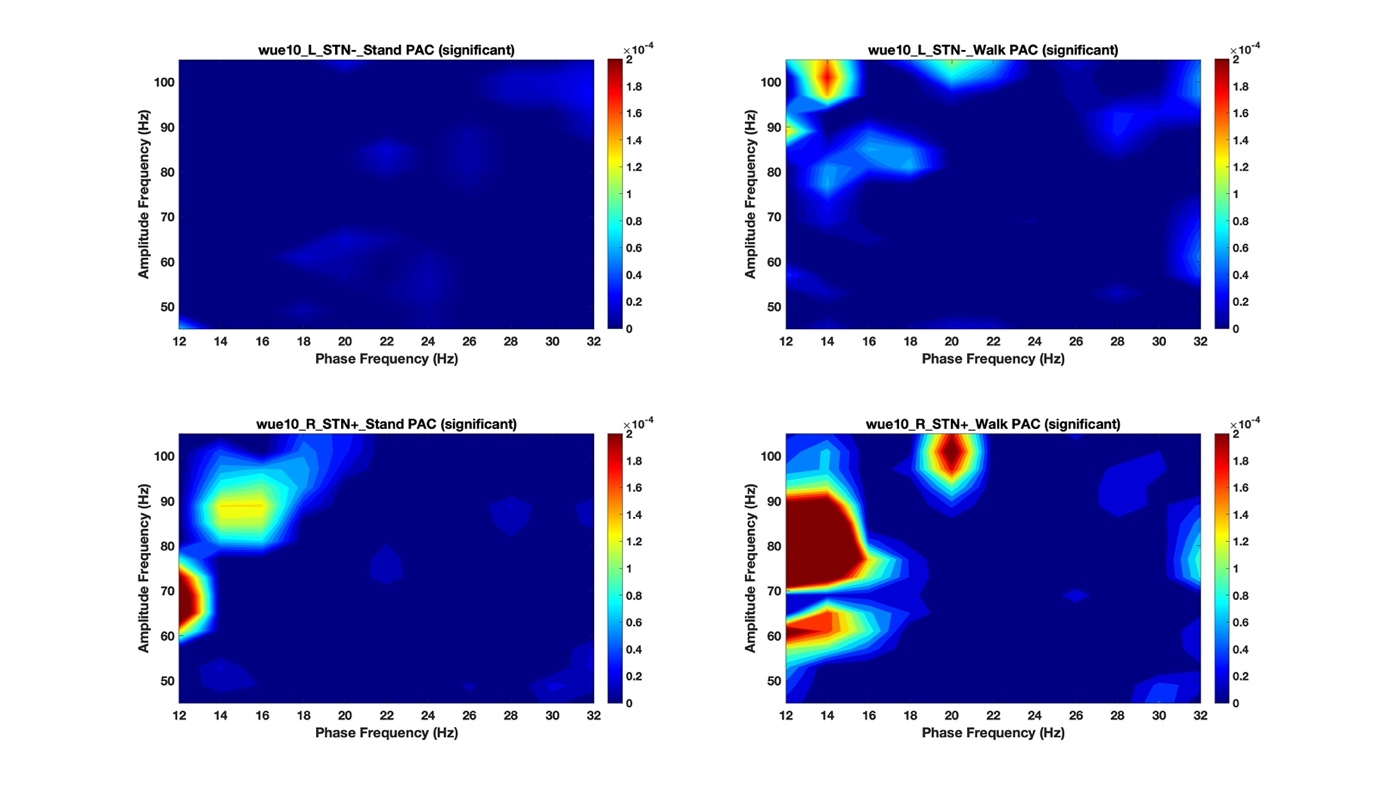


***WUE11***


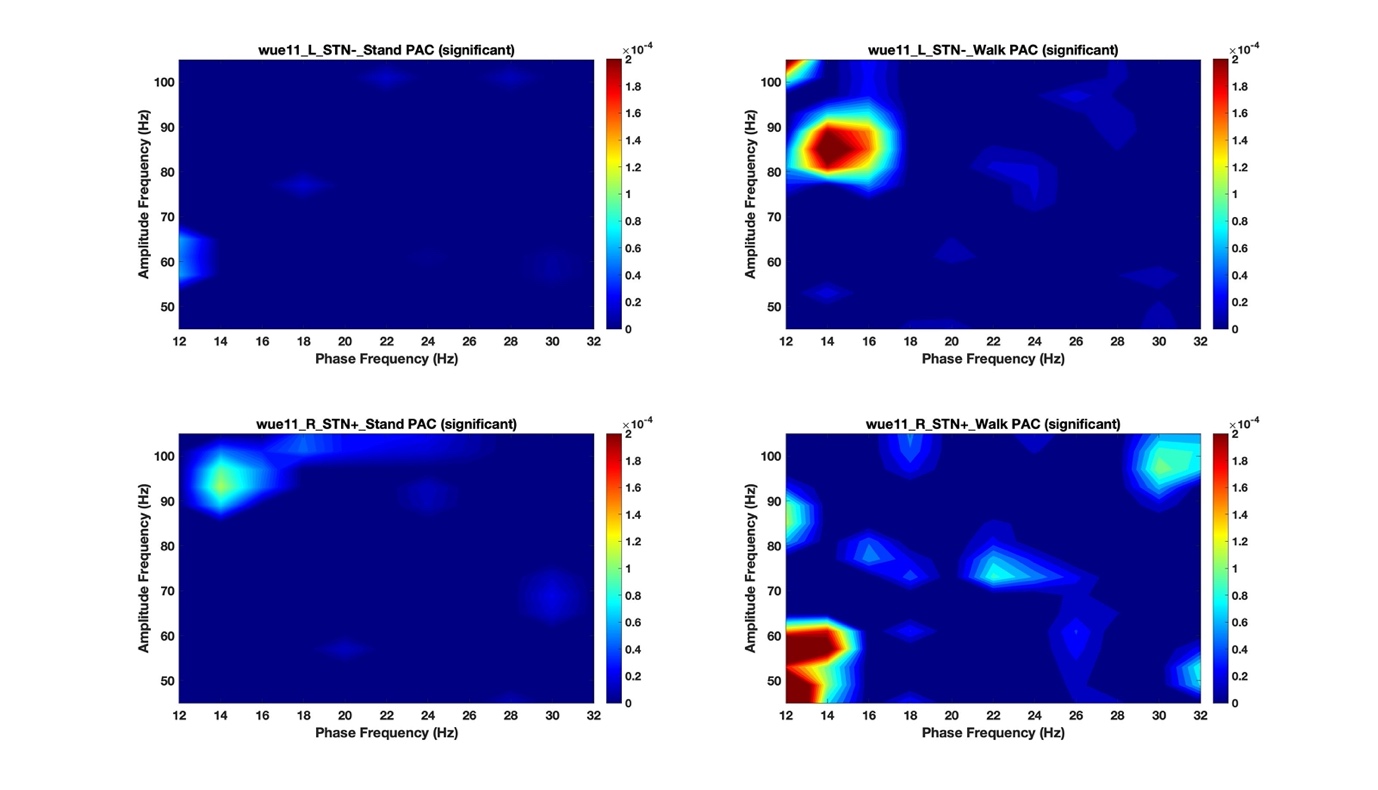

Supplement: Supplementary file 1 — Supplementary Information. [file 41598_2024_57252_MOESM1_ESM.docx]
